# *FTO*-mediated m6A demethylation of *pri-miR-3591* alleviates osteoarthritis progression

**DOI:** 10.1186/s13075-023-03035-5

**Published:** 2023-04-01

**Authors:** Wengang Liu, Tao Jiang, Wei Zheng, Jiayuan Zhang, Anan Li, Chao Lu, Zhaowei Lin

**Affiliations:** 1 Department of Orthopedics, Guangdong Provincial Second Hospital of Traditional Chinese Medicine, Guangzhou, 510095 China; 2 The Fifth Clinical College of Guangzhou, University of Chinese Medicine, Guangzhou, 510405 China; 3 Orthopedics Center, Zhujiang Hospital of Southern Medical University, Guangzhou, 510000 China; 4 Department of Joint and Orthopedics, Zhujiang Hospital of Southern Medical University, Guangzhou, 510000 China

**Keywords:** Osteoarthritis, N6-methyladenosine (m6A), *FTO*, *miR-3591-5p*, *PRKAA2*

## Abstract

**Objectives:**

Increasing evidence have demonstrated the N6-methyladenosine (m^6^A) plays critical roles in osteoarthritis (OA) progression, but the role of m^6^A in OA has not been completely illuminated. Herein, we investigated the function and underlying mechanism of m^6^A demethylase fat mass and obesity-associated protein (*FTO*) in OA progression.

**Materials and methods:**

The *FTO* expression was detected in mice OA cartilage tissues and lipopolysaccharide (LPS)-stimulated chondrocytes. Gain-of-function assays was used to evaluate the role of *FTO* in OA cartilage injury in vitro and in vivo. The miRNA-sequencing, RNA-binding protein immunoprecipitation (RIP), luciferase reporter assay, and in vitro pri-miRNA processing assays were conducted to confirm that *FTO* modulated the *pri-miR-3591* process in an m6A-dependent manner and then the binding sites of *miR-3591-5p* with *PRKAA2*.

**Results:**

*FTO* was outstandingly downregulated in LPS-stimulated chondrocytes and OA cartilage tissues. *FTO* overexpression enhanced the proliferation, suppressed apoptosis, and decreased degradation of extracellular matrix in LPS-induced chondrocytes, whereas *FTO* knockdown contributed to the opposite effects. In vivo animal experiments showed that *FTO* overexpression markedly alleviated OA mice cartilage injury. Mechanically, *FTO*-mediated m6A demethylation of *pri-miR-3591* leaded to a maturation block of *miR-3591-5p*, which relieved the inhibitory effect of *miR-3591-5p* on *PRKAA2* and then promoted the increase of *PRKAA2*, thereby alleviating OA cartilage damage.

**Conclusions:**

Our results attested that *FTO* alleviated the OA cartilage damage by mediating *FTO*/*miR-3591-5p*/*PRKAA2* axis, which provided fresh insights into the therapeutic strategies for OA.

**Supplementary Information:**

The online version contains supplementary material available at 10.1186/s13075-023-03035-5.

## Introduction

Osteoarthritis (OA) is a frequent chronic irreversible joint disease characterized by degradation of cartilage extracellular matrix (ECM) and chondrocytes apoptosis, which influences an increasing number of people around the world [1, 2]. The clinical symptoms of OA patients are joint pain, joint stiffness, joint swelling, and loss of joint function, which may even lead to difficulty in daily activities [3–5]. Currently, non-steroidal anti-inflammatory drugs and surgical intervention are the main treatment measures for OA, but these therapeutic effects are unsatisfactory along with some side effects [6]. Therefore, it is very indispensable to superior elucidate the pathogenesis of OA and explore new therapeutic targets.

N6-methyladenosine (m6A) is a ubiquitous internal modification of eukaryotic RNA, which plays central roles in physiological and pathological processes [7]. The m6A modification has been shown to regulate different stages of RNA metabolism, including maturation, folding, export, translation, and decay, which are regulated by writers protein (METTL3, METTL14, WTAP, and etc.), erasers protein (*FTO*, ALKBH5 and ALKBH3), and readers protein (YTHDCs, YTHDFs, IGF2BPs and etc.) [8, 9]. Previous studies have attested that dysregulation of m^6^A modification and its related genes participate in the development of OA [10–13]. The earliest discovered *FTO* catalyzes m6A demethylation in a Fe(II)—and α-ketoglutarate-dependent manner [14]. The previous research has shown that *FTO* polymorphism rs8044769 alleles is a risk factor for temporomandibular joint OA, which may be involved in temporomandibular joint OA progression [15]. Nevertheless, the specific roles and potential mechanisms of *FTO* in OA has not been explored.

MicroRNAs (miRNAs) are a class of small non-coding single-stranded RNAs with a length of about 22 nucleotides encoded by endogenous genes, which play important roles in joint homeostasis and the pathogenesis of OA [16]. Recently, more and more attention has been paid to the study that m6A modification regulates miRNAs maturation and then affects target gene expression [17, 18]. For examples, METTL3 accelerated the miR-221/222 maturation by mediating m6A modification, thereby resulting in the inhibition of PTEN [19]. Moreover, *FTO* outstandingly reduced the miR-576 formation by mediating m6A modification and then contributed to the enhancement of CDK6, thus promoting bladder cancer progression [20]. *FTO* significantly increased the ARL5B expression through the suppression of miR-181-3p, thus promoting the invasion and migration of breast cancer cells [21]. *FTO* knockdown regulated m6A-mediated pri-miR-10a processing to promote miR-10 maturation, and then inhibited MTMR3 expression, thereby promoting glioma progression [22]. These studies have disclosed that *FTO* plays a crucial part in multiple tumors by regulating the m6A modification of miRNAs. However, whether *FTO* is involved in the development of OA remains unclear.

In this study, our founding unmasked that *FTO* ameliorated OA cartilage damage in vitro and in vivo, whereas *FTO* knockdown aggravated LPS-induced chondrocytes injury in vitro. Moreover, this study revealed that *FTO*-mediated m^6^A demethylation of *pri-miR-3591* leaded to a maturation block of *miR-3591-5p*, which resulted in the increase of *PRKAA2* expression, thereby inhibiting chondrocytes damage. Briefly, our founding not only revealed a novel mechanism of chondrocytes injury in OA, but also demonstrated that *FTO* might be a promising therapeutic target for patient with OA.

## Material and methods

### Human articular chondrocytes culture

As previously described [23], human articular chondrocytes were separated from articular cartilage gained from OA patient undergoing total knee replacement surgery (*n* = 6). Briefly, fresh articular cartilage tissues were collected and digested with 0.25% trypsin (Sigma-Aldrich). After digestion at 37 °C for 30 mim to remove fibroblasts, these articular cartilages were digested with 0.2% type II collagenase (Sigma-Aldrich) at 37 °C for 6 h and then filtered by a 100-μm cell strainer. Finally, the cells collected by centrifugation (1000 rpm/mim, 10 min) were cultured in Dulbecco’s modified Eagle’s medium/nutrient mixture F12 (DMEM/F12, Thermo Fisher Scientific) containing 10% fetal bovine serum (FBS, Gibco) in the constant temperature incubator with 5% CO_2_ and then observed the cell morphology under the microscope. The cells cultured to the second generation were identified as chondrocytes by immunofluorescence (*Collagen II* antibody) in addition to observing the cells morphology, and the chondrocytes cultured to the third generation were used for later experimental research. In addition, all clinical samples in this study were confirmed by the Ethics Committee of Zhujiang Hospital of Southern Medical University (No.2018-GJGBWK), and all participants signed written informed consent prior to the program.

### Adenovirus construction and LPS stimulation

The adenovirus of *FTO* or *PRKAA2* overexpression / knockdown and their negative control were constructed by Genechem (Nanjing, China). The adenovirus particles of *miR-3591-5p* mimics, *miR-3591-5p* inhibitor, and their negative controls were gained from Ribobio (Guangzhou, China). All cells were infected with the adenovirus on the basis of the instructions, and the infection efficiency was verified by quantitative real-time PCR (RT-qPCR) or western blotting. After 48 h of adenovirus transmission, chondrocytes were stimulated with lipopolysaccharide (LPS; Sigma-Aldrich) for the 24 h and then used for further studies. The sequences of all *shRNAs*, *miR-3591-5p* mimics, *miR-3591-5p* inhibitor, and their negative controls are showed in Supplementary Table 1.

### RT-qPCR

Total RNAs were extracted from chondrocytes or articular cartilage by the TRIzol reagent (Takara, Japan), and then was reversed transcribed into complementary DNA by PrimeScript™ RT reagent Kit with gDNA Eraser (#RR047A, Takara). Finally, RT-qPCR amplification was performed (Roche, Indianapolis, IN, USA) on the Roche LightCycler ®96 Real-Time PCR System using BenyoFast^TM^SYBR Green qPCR Mix (#D7260, Beyotime). Small RNA RNU6 (U6) and Glyceraldehyde-3-phosphate dehydrogenase (GAPDH) was served as an internal reference for *miR-3591-5p* and other mRNAs, respectively. All genes expression levels was evaluated by 2^−ΔΔCt^ method. All primers sequences involved in RT-qPCR were gained from Sangon Biotech (Shanghai, China) and displayed in Supplementary Table 2.

### RNA m^6^A quantification

Total RNAs were separated from chondrocytes or articular cartilage tissues using TRIzol reagent (Takara, Japan), and then relative RNA m6A levels were quantified by m6A RNA methylation assay kit (colorimetric; Abcam, ab185912) according to the instructions. Finally, the relative m6A RNA methylation levels were computed by the following formula: m6A% = [(Sample OD-NC OD)/S] × 100%/[(PC OD-NC OD)/P], where the OD indicated absorbance value at 450 nm, NC indicated negative control, PC indicated positive control, S indicated the amount of input sample RNA, and P indicated the amount of input positive control.

### Western blot

Total proteins were separated from chondrocytes and articular cartilage using pre-cooled radioimmunoprecipitation buffer (Beyotime, Haimen, China) containing protease inhibitor (Roche, Indianapolis, IN, USA). After proteins quantification and denaturation, equal quantity of proteins were isolated using electrophoresis and then transferred into a polyvinylidene difluoride membranes (Millipore, USA). Subsequently, this membrane was blocked by 5% skim milk for 1 h, and then incubated with primary antibodies overnight at 4 °C. After washing the membrane, the membrane was incubated with secondary antibody (Abcam, UK) for 2 h at room temperature. Finally, the protein band was visualized with the ECL regents (DINGGUO Biology, China) on Gel imaging system (GE Healthcare). In addition, the gray values of each band were analyzed using the Image J software. Primary antibodies in the study are as follows: *FTO* (ab126605, Abcam; 1:1000), *ADAMTS5* (ab41037, Abcam; 1:1000), *MMP13* (ab219620, Abcam; 1:1000), *Collagen II* (ab34712, Abcam; 1:1000), *Aggrecan* (ab3778, Abcam; 1:1000), *PRKAA2* (ab126911, Abcam; 1:1000), and *GAPDH* (ab181602, Abcam; 1:2000).

### Cells proliferation

The cell counting kit-8 (CCK-8) assay and a 5-ethynyl-20-deoxyuridine (EDU) assay were applied to analyze cell proliferation. For CCK-8 assay, the treated cells were dealt with Enhanced Cell Counting Kit-8 (Beyotime, #C0042) according to the instructions. The absorbance of each sample was measured by a microplate reader (Tecan, F50) at 450 nm. For EDU assay, the treated cells were conducted by EDU assay kit (Ribobio, Guangzhou, China) on the basis of the instructions. After nuclear staining using Hoechst 33,342, the images were obtained by an inverted fluorescence microscope (Mshot, MF52) and then analyzed using image J.

### Cells apoptosis

Cells apoptosis were estimated via flow cytometry and *Caspase-3* activity. For flow cytometry, the treated chondrocytes were performed using Annexin V-FITC/PI double-staining kit (Abcam) in the light of the instructions. The treated chondrocytes were blended with Annexin V-FITC dye and PI dye and co-incubated for 20 min at room temperature in avoid light. Finally, the apoptosis rate was detected and analyzed by BD FACSVerse™ Flow Cytometer (BD Biosciences, USA) and Cell Quest (BD Bioscience, USA) software. In addition, the *caspase-3* activity was measured using the *Caspase-3* Colorimetric Assay Kit (Keygen Biotech, Nanjing, China) in the light of the manufacturer’s protocol. The absorbance of each sample was detected by a microplate reader (Tecan, F50) at 450 nm.

### miRNA sequencing and data analysis

The miRNA sequencing and data analysis were carried out by Shanghai Biotechnology Corporation (Shanghai, China). In short, total RNAs were separated from each sample using mirVana™ miRNA Isolation Kit (Cat #. AM1561, Austin TX, US) following the manufacturer’s standard operating procedures and then got through the electrophoresis quality inspection by Agilent 2100 Bioanalyzer (Agilent Technologies, Santa Clara, USA) for subsequent detection. Subsequently, the sequencing sample library construction and cluster generation were performed. After the sequencing sample library construction and cluster generation were completed, the flow cell carrying the cluster was sequenced on the machine. The sequencing process was commanded by the data collection software provided by Illumina, and real-time data analysis was carried out. The differentially expressed miRNAs were identified with *p* < 0.05 and |log_2_fold-change (FC)|> 2.

### Data sources and bioinformatics analysis

A sequence-based m^6^A modification site predictor (SRAMP) was used for predicting m^6^A modification sites on *pri-miR-3591*. The mRNAs expression profile related to OA progression was investigated in GSE113825 and GSE16464 datasets from Gene Expression Omnibus (GEO) database and analyzed using GEO2R. The threshold criteria for differential expressed genes were log_2_FC ≥  − 1.5 and *p* value < 0.01. The potential targets genes of *miR-3591-5p* were forecast through miRDB (http://mirdb.org/) and TargetScan (http://www.targetscan.org/), and then these predicted mRNAs were crossed with those mRNAs that significantly downregulated in GSE113825 and GSE16464 dataset.

### RNA immunoprecipitation (RIP) analysis

The EZ-Magna RIP™ RNA-Binding Protein Immunoprecipitation Kit (Millipore) was applied to execute RIP experiment. Briefly, the treated cells were lysed in RIP lysis buffer, and then these lysate was incubated with magnetic beads coupled with the anti-*Ago2* antibody (ab186733, Abcam), anti-*DGCR8* antibody (ab191875, Abcam), anti-*m*^*6*^*A* antibody (ab286164; Abcam), or Immunoglobulin G antibody (*IgG*, ab6715, Abcam) at 4 °C for 6 h. After washing, co-precipitated RNAs were extracted from magnetic beads complex using TRIzol reagent (Takara, Japan) and then RT-qPCR was performed using specific primers.

### In vitro* pri-miRNA processing assays*

In vitro *pri-miR-3591* processing assays was carried out according to the methods previously reported [24, 25]. In brief, the mutant *pri-miR-3591*[m^6^A] were generated by replacing adenosine with thymine, and then *pri-miR-3591*[m^6^A]-WT or *pri-miR-3591*[m^6^A]-Mut and *pri-miR-1–1* (control) were incubated with whole cellular lysates of chondrocytes co-overexpressing *DROSHA* and *DGCR8*. Finally, total RNAs extracted from reaction products was subjected to RT-qPCR analysis.

### Luciferase reporter assay

To further validate the effect of m6A modification on *miR-3591-5p* expression, m^6^A modification sites on *pri-miR-3591* sequences were predicted through SRAMP. We identified one putative m6A recognition sites on *pri-miR-3591* sequences. According to this predicted results, mutagenesis from adenosine to thymine was generated by QuikChange II Site-Directed Mutagenesis Kit (Agilent, USA) in the light of the instruction, and then the wild-type and mutant *pri-miR-3591* reporter vectors were co-transfected with *shFTO* plasmid into chondrocytes. Transfer after 48 h, luciferase activities were assessed by a Dual Luciferase Assay Kit (Promega, Madison, WI, USA) in accordance with the instructions. For the verification of *miR-3591-5p* and *PRKAA2* targeted interactions, the wild-type or mutant-type of *PRKAA2* (containing the binding site of *miR-3591-5p*) was cloned into the luciferase vector, and then transfected into in chondrocytes together with *miR-3591-5p* mimics or NC mimics, respectively. Transfer after 48 h, luciferase activities were assessed by a Dual Luciferase Assay Kit (Promega, Madison, WI, USA) on the base of the instructions. Finally, the signal value of Renilla luciferase was normalized as an internal reference.

### Destabilizing the medial meniscus (DMM)-induced OA mouse model

All animal experiments were executed in the light of the Guide for the Care and Use of Laboratory Animals and confirmed by the Animal Care Committee of Zhujiang Hospital of Southern Medical University (No: 2018-GJGBWK). The male C57BL/6 mice (SPF, 8-week-old) were randomly divided into four groups: sham (*n* = 6), model (*n* = 6), model + adeno-associated virus-negative control** (**AVV-NC**)** (*n* = 6), and model + AVV-*FTO* groups (*n* = 6). After acclimating for one week, mice in other groups were treated with DMM surgery to induce OA model as previously described except for the sham groups [26]. Mice in sham groups was underwent only the skin of the right knee joint incision. The AAV-NC and AAV-*FTO* were constructed by HANBIO (Shanghai, China). The mice in model + AAV-NC and model + AAV-*FTO* groups were intra-articularly injected with 10 μL of AAV-NC and AAV-*FTO* (1 × 10^13^ vg/ml) through the medial parapatellar area at 2 weeks after the DMM operation, respectively. At the same point in time, the mice in the sham and model groups were intra-articularly injected with the equal volume of normal saline. After 8 weeks post-surgery, knee joints were harvested from euthanized mice for later histological analysis and molecular analysis.

### Histological analysis

The fresh knee joints were fixed with 4% paraformaldehyde, decalcified in 15% EDTA-2Na, dehydrated in gradient ethanol, embedded in paraffin, and then sliced into 5-μm sections. After dewaxing in xylene and hydration with a graded ethanol series, the sections were stained with safranin-O/fast green (Yeasen) and hematoxylin and eosin (HE, Sigma-Aldrich), respectively. The images of each section were visualized and photographed under an inverted fluorescence microscope (Mshot, MF52). In addition, the degree of articular cartilage damage in OA mice was assessed by Osteoarthritis Research Society International (OARSI) score as described previously [27].

### Statistical analysis

All data in present research are indicated as the mean ± standard deviation (SD). The GraphPad Prism 8.0 software (GraphPad, San Diego, CA, USA) was applied to perform statistical analysis and draw diagram. Differences between groups were analyzed using Student’s *t*-test or two-way ANOVA. The correlation between *FTO* mRNA expression and total m6A levels was evaluated by Pearson correlation coefficient. A *p*-value < 0.05 was considered statistically significant and each experiment was carried out in triplicate.

## Results

### Downregulation of *FTO* expression in OA cartilage and LPS-stimulated chondrocytes

**Fig. 1:**
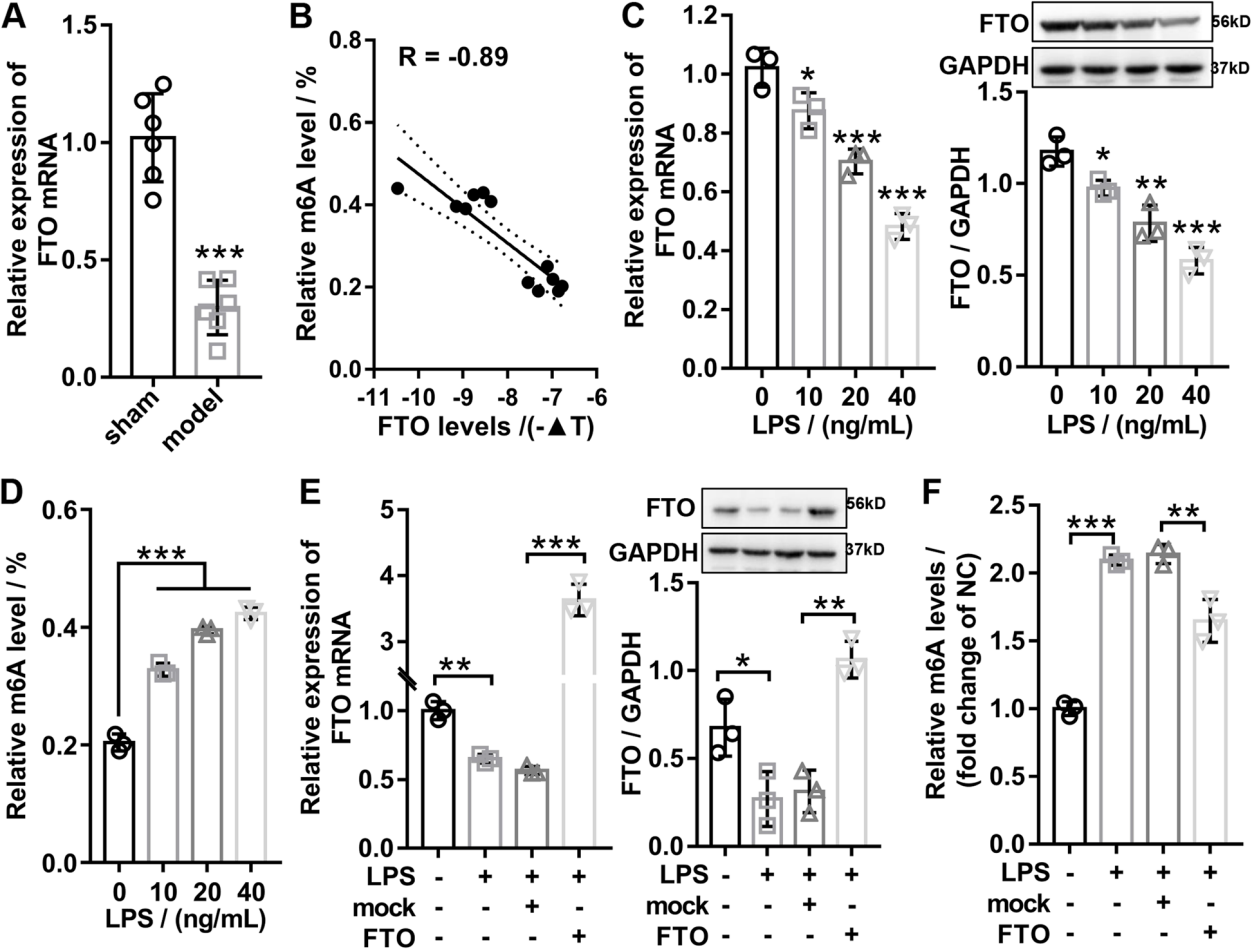
Downregulation of *FTO* in OA and LPS-stimulated chondrocytes. **A** RT-qPCR results attested that the mRNA expression of *FTO* was significantly downregulated in the cartilage tissues of OA mice. **B** Pearson correlation coefficient analysis certified that the mRNA expression of *FTO* was markedly negatively related with the total m^6^A levels in cartilage tissue of OA mice (*R* =  − 0.89). **C** After the chondrocytes were dealt with different concentration gradients of LPS, the mRNA and protein expression of *FTO* was evaluated by RT-qPCR and western blotting. **D** After the chondrocytes were dealt with different concentration gradients of LPS, the relative m^6^A levels were detected by the m^6^A RNA methylation assay kit. **E** RT-qPCR and western blot were applied to confirm the overexpression of *FTO* in LPS-treated chondrocytes. **F** The m^6^A levels were detected using m.^6^A RNA methylation assay kit in different groups. Sham, mice without DMM; model, DMM surgery-induced OA mice; LPS, lipopolysaccharide; *FTO*, *FTO* overexpression adenovirus; mock, negative control corresponding to *FTO* overexpression adenovirus; *n* = 3 ~ 6, **p < 0.01, ***p < 0.001

To confirm the expression of *FTO* in cartilage tissues of OA, the expression of *FTO* were detected by RT-qPCR in cartilage tissues of mice with or without OA. RT-qPCR analysis revealed that the *FTO* was significantly downregulated in the cartilage tissues of the mice with OA compared with the mice without OA (Fig. 1A). Then, we used RNA m6A colorimetry to detect the total m^6^A levels in RNA extracted from cartilage tissues of mice with or without OA, and correlation analysis indicated that *FTO* mRNA expression was markedly negative correlated with the total m^6^A levels in OA cartilage (Fig. 1B). Subsequently, we also studied the changes of *FTO* expression and m6A levels under the LPS concentration gradient in the OA cell model and found that the expression of *FTO* significantly decreased with the increase of LPS concentration in a concentration dependent manner (Fig. 1C), whereas the m^6^A levels significantly enhanced with the LPS concentration increasing (Fig. 1D). Further overexpression of *FTO* in LSP-induced OA chondrocytes was confirmed by western blotting and RT-qPCR (Fig. 1E) and found that the *FTO* overexpression significantly suppressed the total m6A levels induced by LPS (Fig. 1F). Taken together, our findings showed that *FTO* was outstandingly downregulated in OA cartilage tissues and LPS-stimulated chondrocytes, and the *FTO* expression was significantly negative correlated with the total m^6^A levels.

### Effects of *FTO* on LPS-induced chondrocyte damage in vitro

**Fig. 2:**
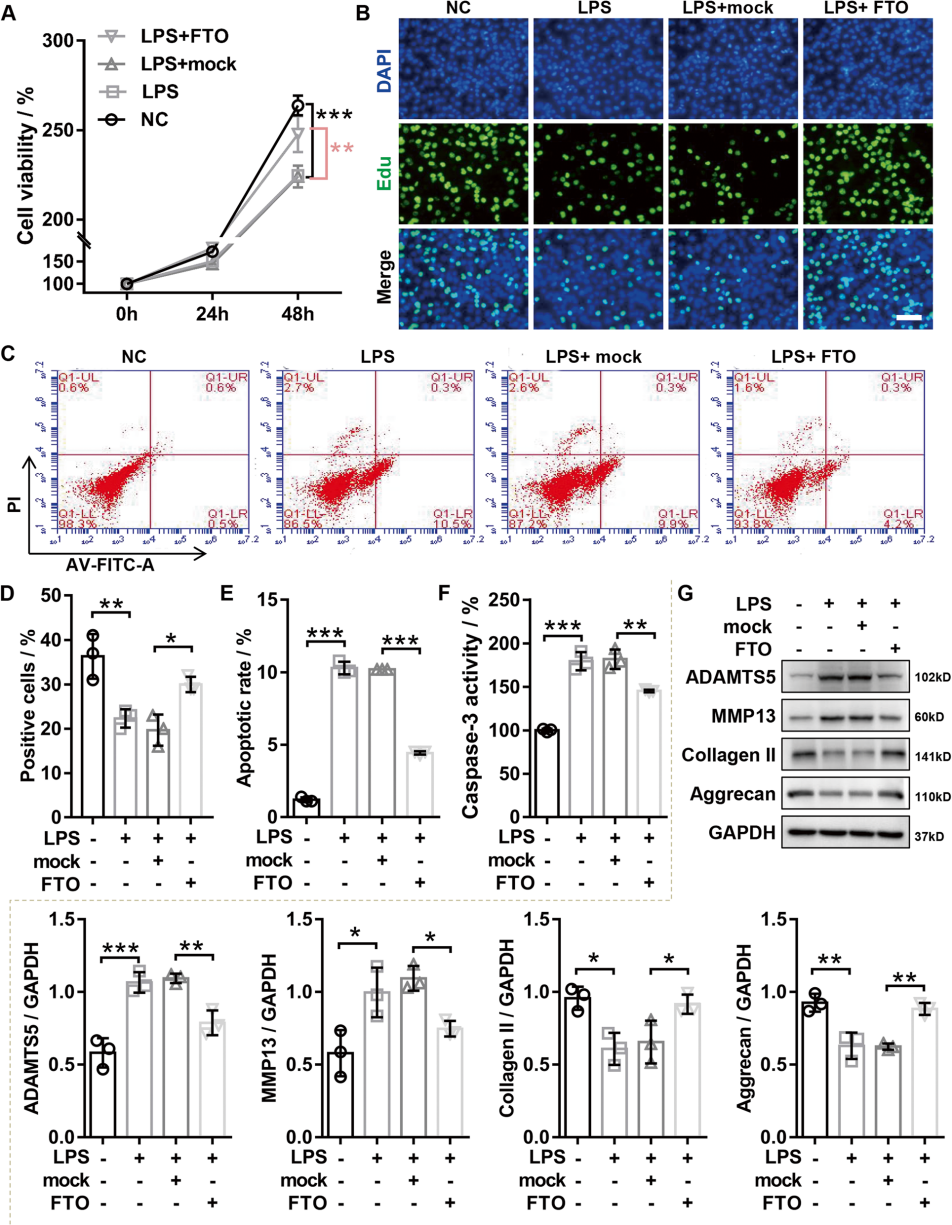
*FTO* overexpression aggravated alleviated LPS-induced chondrocyte damage in vitro. Chondrocytes were transfected with *FTO* or mock and then treated with 40 ng/mL LPS for 24 h. **A** CCK-8 was applied to evaluate the viability of chondrocytes. **B** and **D** EDU staining was applied to measure the proliferation ability of chondrocytes. **C** and **E** The apoptosis of chondrocytes was evaluated by flow cytometry. **F** The caspase-3 activity was evaluated by caspase-3 activity kit. **G** Western blotting was applied to assess the expression of *MMP13*, *ADAMTS5*, *Aggrecan*, and *COL2A1*. LPS, lipopolysaccharide; *FTO*, *FTO* overexpression adenovirus; mock, negative control corresponding to *FTO* overexpression adenovirus; *n* = 3, *p < 0.05, **p < 0.01, ***p < 0.001

To explore the roles of *FTO* in chondrocytes, the chondrocytes with or without overexpressing *FTO* were treated with or without LPS for 24 h. CCK-8 results indicated that LPS treatment remarkably reduced cell viability, while *FTO* overexpression reversed this process (Fig. 2A). Consistently, EDU experiments also showed that *FTO* overexpression significantly reversed the inhibition of LPS on chondrocyte proliferation (Fig. 2B and D). In addition, flow cytometry data indicated that *FTO* overexpression significantly impaired the elevated levels of apoptosis induced by LPS treatment (Fig. 2C and E). Consistent with flow cytometry, we found that upregulation of *FTO* dramatically reduced the LPS-induced caspase-3 activity in chondrocyte (Fig. 2F). Since the degradation of articular cartilage is the main clinical symptoms of OA, western blot was used for detecting the ECM degradation-related protein expression. The results attested that *FTO* overexpression inhibited the expression of major cartilage-degrading metalloproteinase (*MMP-13* and *ADAMTS-5*) and enhanced the expression of chondrogenic markers (*Collagen II* and *Aggrecan*) in LPS-treated chondrocytes (Fig. 2G). In addition, the *FTO* knockdown was confirmed in LSP-induced OA chondrocytes using western blotting and RT-qPCR (Supplementary Fig. 1A). The results of CCK-8 and EDU assay attested that downregulation of *FTO* significantly aggravated the inhibition of LPS on chondrocyte proliferation (Supplementary Fig. 1B and C). Next, we assessed the effects of *FTO* knockdown on chondrocyte apoptosis. Flow cytometric analysis revealed that the cell apoptosis in LPS-induced chondrocytes was markedly increased following *FTO* downregulation (Supplementary Fig. 1D and F). And consistent with the trend of flow cytometry, the results of caspase-3 activity detection indicated that downregulation of *FTO* notably promoted the LPS-induced caspase-3 activity in chondrocytes (Supplementary Fig. 1E). In addition, the results of western blotting attested that *FTO* knockdown outstandingly enhanced the major cartilage-degrading metalloproteinase (*MMP-13* and *ADAMTS-5*) protein expression and repressed the protein expression of chondrogenic markers (*Collagen II* and *Aggrecan*) in LPS-treated chondrocytes (Supplementary Fig. 1G), suggesting that knockdown of *FTO* significantly inhibited the chondrogenic capacity of chondrocytes. Taken together, our data suggested that overexpressing *FTO* promoted proliferation, suppressed apoptosis, and ameliorated ECM degradation in LPS-induced chondrocyte, whereas knockdown of *FTO* suppressed the proliferation, promoted the apoptosis, and aggravated ECM degradation in LPS-induced chondrocytes.

### *FTO* regulated the *miR-3591-5p* maturation in an m^6^A-dependent manner base on miRNA-Seq analysis

**Fig. 3:**
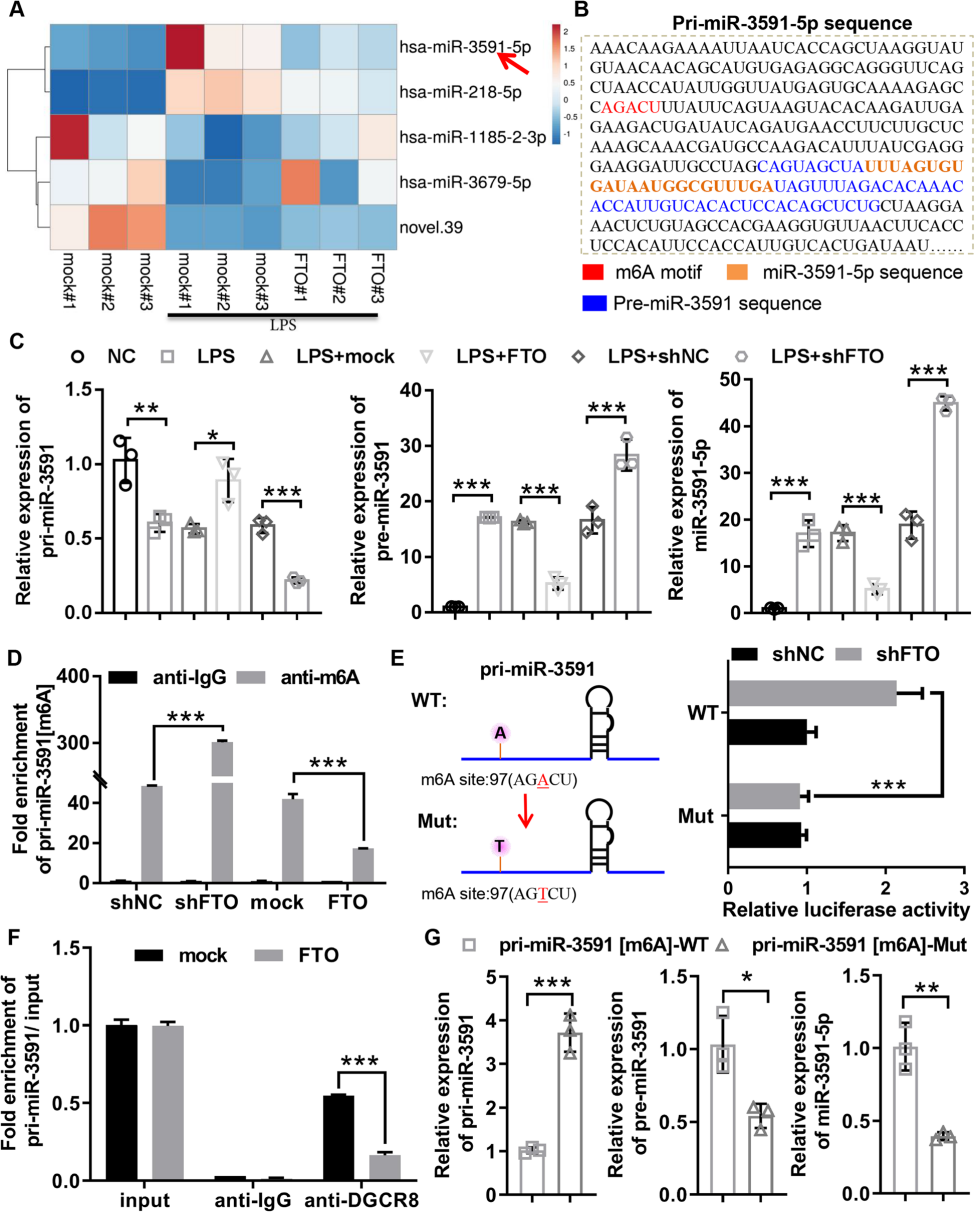
*FTO* regulated the *miR-3591-5p* maturation in an m^6^A-dependent manner base on miRNA-Seq analysis. **A** Heatmap showed miRNAs expression profile between the mock group, LPS + mock group and LPS + *FTO* group by miRNA-seq analysis. **B** The sequences of *pre-miR-3591*, *miR-3591-5p*, and the potential m6A motif (AGACU) splicing site were highlighted with different colors. **C** Chondrocytes were transfected with sh*FTO* or shNC or mock or *FTO*, and then dealt with 40 ng/mL LPS for 24 h. RT-qPCR was used to assess the mRNA expression *pri-miR-3591*, *pre-miR-3591*, and *miR-3591-5p*. **D** Chondrocytes were transfected with sh*FTO* or shNC or mock or *FTO*, and then, the *pri-miR-3591* [m^6^A] levels was evaluated using MeRIP coupled with RT-qPCR analysis. **E** Relative luciferase activity of the wild-type and mutant-type *pri-miR-3591* reporter vectors were assessed in *FTO* knockdown chondrocytes. **F** RIP combined with RT-qPCR was applied to analyze the *pri-miR-3591* binding to *DGCR8* in chondrocytes with or without overexpressing *FTO*. **G** Quantification of *miR-3591-5p*, *pre-miR-3591*, and *pri-miR-3591* in the reaction mixture was detected by RT-qPCR, and the results indicated that mutation of [m.^6^A] *pri-miR-3591* abolished *pri-miR-3591* processing in the in vitro reaction system. LPS, lipopolysaccharide; *FTO*, *FTO* overexpression adenovirus; mock, negative control corresponding to *FTO* overexpression adenovirus; sh*FTO*, *FTO* knockdown adenovirus; shNC, negative control corresponding to sh*FTO* adenovirus; *n* = 3, *p < 0.05, **p < 0.01, ***p < 0.001

To investigate the molecular mechanism of *FTO* in cartilage injury of OA, we identified miRNAs regulated by *FTO* using miRNA-sequencing analysis. We found that two significantly downregulated miRNAs (*hsa*-*miR-3591-5p* and *hsa-miR-218-5p*) and three markedly upregulated miRNAs (*hsa-miR-1185–2-3p*, *hsa-miR-3679-5p*, and *novel.39*) in LPS-treated chondrocytes were reversed by *FTO* overexpression (Fig. 3A). Since the decreased m^6^A levels leads to the arrest of pri-miRNA processing, we selected miRNAs (hsa-*miR-3591-5p* and *hsa-miR-218-5p*) negatively associated with *FTO* and predicted m^6^A modification sites on *pri-miR-3591* and *pri-miR-218* RNA sequences using SRAMP. The results showed that an AGACU m^6^A sequence motif in RNA sequences of *pri-miR-3591* (Fig. 3B), whereas the site was not found in *pri-miR-218*. So *miR-3591-5p* was selected for further study. Further RT-qPCR analysis confirmed that pri-miRNA-3591 was significantly accumulated in *FTO*-overexpressing chondrocytes and remarkably reduced in *FTO*-inhibiting chondrocytes, while opposite results were obtained for the expression of *pre-miR-3591* and *miR-3591-5p* (Fig. 3C), suggesting that *FTO* might regulate the *miR-3591-5p* maturation. To further investigate the effect of m^6^A modification on *pri-miR-3591* processing, the MeRIP coupled with RT-qPCR was used to detect the enrichment level of *pri-miR-3591* in *FTO* overexpression or knockdown chondrocyte and found that overexpression of *FTO* dramatically decreased *pri-miR-3591* m^6^A levels, while knockdown of *FTO* markedly increased *pri-miR-3591* m^6^A levels (Fig. 3D), while the *pri-miR-3591* wild type dual-luciferase reporter (containing potential m6A sites, *pri-miR-3591* [m^6^A]-WT) and *pri-miR-3591* mutant type dual-luciferase reporter (the adenosine base in m^6^A consensus sequences replaced with thymine to abolish the m^6^A modification, *pri-miR-3591* [m^6^A]-Mut) was transfected into chondrocytes with or without *FTO* knockdown for the dual-luciferase reporter assay, and the results revealed that the luciferase activity of the wild-type *pri-miR-3591*[m^6^A] was significantly increased by *FTO* knockdown, but the luciferase activity of the mutant-type *pri-miR-3591*[m^6^A] did not effect by *FTO* knockdown, indicating the *pri-miR-3591* processing was modulated by *FTO*-associated m^6^A modification (Fig. 3E). To further confirm a direct role of m^6^A in *miR-3591-5p* maturation, we carried out RIP with *DGCR8* antibody and in vitro RNA processing assays. The RIP with *DGCR8* antibody followed by RT-qPCR demonstrated that the *DGCR8*-bound *pri-miR-3591* expression remarkably decreased in chondrocytes with *FTO* overexpression compared with that in chondrocytes without *FTO* overexpression (Fig. 3F). We further executed in vitro RNA processing assays using in vitro transcribed *pri-miR-3591*[m^6^A]-WT or *pri-miR-3591* [m^6^A]-Mut and whole cell lysates of chondrocytes with *DGCR8* and *DROSHA* overexpression. RT-qPCR analysis indicated that the processing rate of pri-miR-3951 to *pre-miR-3591* and *miR-3591-5p* in *pri-miR-3591*[m^6^A]-Mut was outstandingly decreased compared with that of in *pri-miR-3591*[m^6^A]-WT (Fig. 3G). Collectively, the above results demonstrated that *FTO* regulated the *miR-3591-5p* maturation in an m^6^A-dependent manner.

### *miR-3591-5p* aggravated LPS-induced chondrocytes damage in vitro by targeting regulation of *PRKAA2*

**Fig. 4:**
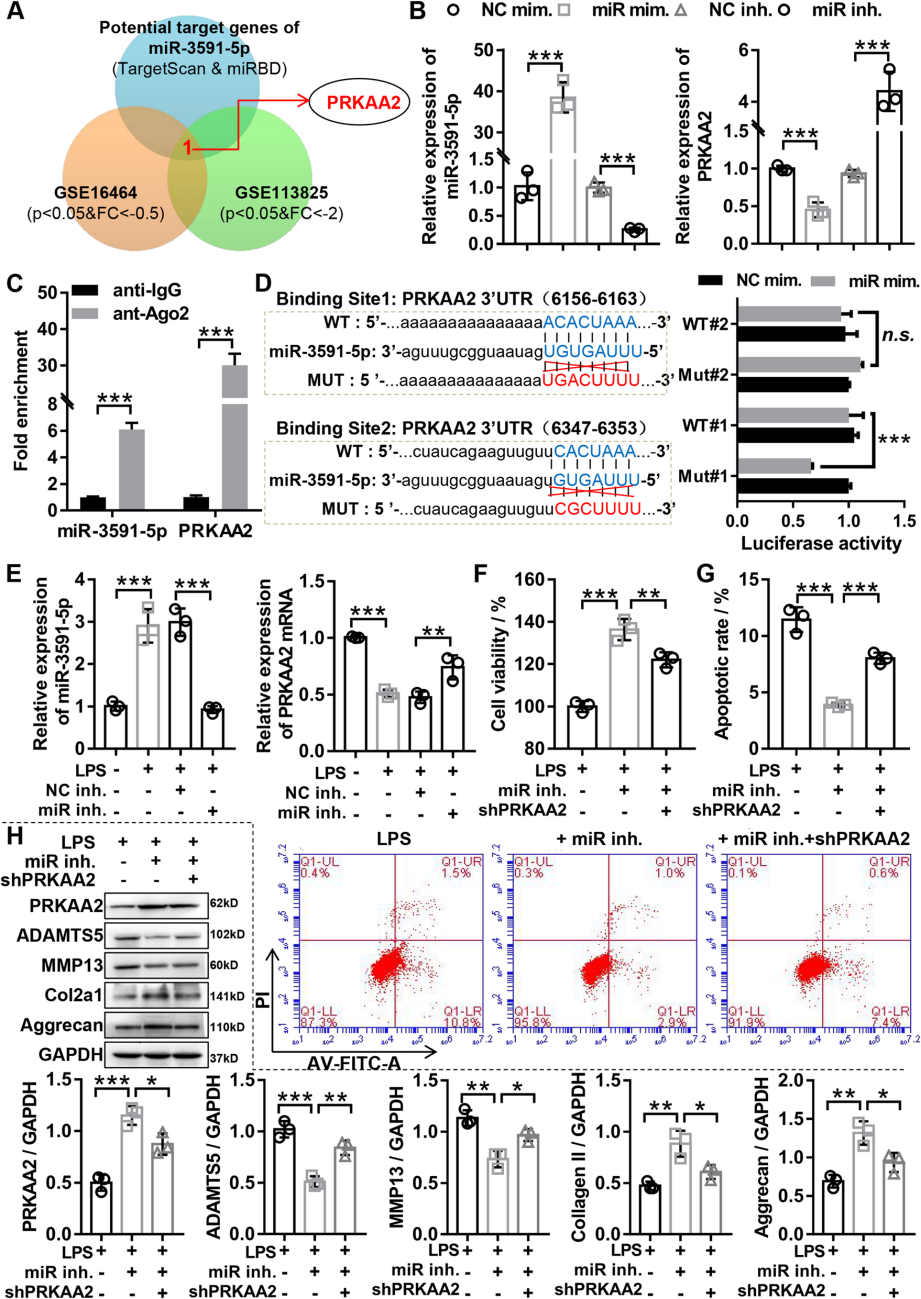
*miR-3591-5p* aggravated LPS-induced chondrocytes damage in vitro by targeting regulation of *PRKAA2*. **A** Common target genes predicted by TargetScan and miRBD were intersected with the significantly downregulated mRNA in OA cartilage from GSE113825 and GSE16464 analysis. **B** The expression levels of *miR-3591-5p* and *PRKAA2* were tested by RT-qPCR in chondrocytes with *miR-3591-5p* mimics or inhibitor. **C** RIP-Ago2 analysis indicated that interaction between *miR-3591-5p* and *PRKAA2*. **D** Interaction between *miR-3591-5p* and *PRKAA2* was affirmed by the dual-luciferase reporter assay. **E** Chondrocytes were transfected with miR-3951 inhibitor or NC inhibitor, and then treated with 40 ng/mL LPS for 24 h. RT-qPCR was used to detect the mRNA expression *miR-3591-5p* and *PRKAA2*. **F** Chondrocytes were transfected with miR-3951 inhibitor or a combination of miR-3951 inhibitor and sh*PRKAA2* and then treated with 40 ng/mL LPS for 24 h. CCK-8 was used to detect the viability of chondrocytes. **G** Chondrocytes were transfected with miR-3951 inhibitor or a combination of miR-3951 inhibitor and sh*PRKAA2* and then treated with 40 ng/mL LPS for 24 h. The apoptosis of chondrocytes was tested by flow cytometry. **H** Chondrocytes were transfected with miR-3951 inhibitor or a combination of miR-3951 inhibitor and sh*PRKAA2* and then treated with 40 ng/mL LPS for 24 h. Western blotting was applied to assess the expression of *MMP13*, *ADAMTS5*, *Aggrecan*, and *COL2A1*. LPS, lipopolysaccharide; NC mim., negative control corresponding to *miR-3591-5p* mimics adenovirus; miR mim., *miR-3591-5p* mimics adenovirus; NC inh., negative control corresponding to *miR-3591-5p* inhibitor adenovirus; miR inh., *miR-3591-5p* inhibitor adenovirus; sh*PRKAA2*, *PRKAA2* knockdown adenovirus; *n* = 3, *p < 0.05, **p < 0.01, ***p < 0.001

To investigative the regulatory mechanism of *miR-3591-5p* in OA, the common target mRNAs of *miR-3591-5p* predicted by TargetScan and miRBD were intersected with the significantly downregulated mRNA in OA cartilage from GSE113825 and GSE16464 analysis, uncovering that *PRKAA2* might be a potential target gene of *miR-3591-5p* (Fig. 4A). After treatment of cells with mimic or inhibitor of *miR-3591-5p*, the results of RT-qPCR unmasked that overexpressing *miR-3591-5p* markedly downregulated *PRKAA2* expression, whereas inhibition of *miR-3591-5p* outstandingly upregulated *PRKAA2* expression, suggesting that *PRKAA2* was negatively regulated by *miR-3591-5p* (Fig. 4B). Then, the interaction between *PRKAA2* and *miR-3591-5p* was confirmed by RIP and a dual-luciferase reporter assay. The RIP with Ago2 antibody followed by RT-qPCR uncovered that the expression of *PRKAA2* and *miR-3591-5p* were significantly increased in the miRNA complex (Fig. 4C). In addition, the prediction results showed that *PRKAA2* had two potential binding sites with *miR-3591-5p*, and the dual-luciferase reporter assay revealed that the luciferase activity of *PRKAA2*-WT#1 was significantly decreased by *miR-3591-5p* overexpression, but the luciferase activity of *PRKAA2*-Mut#1, *PRKAA2*-WT#2, and *PRKAA2*-Mut#2 did not effect by the overexpression of *miR-3591-5p* (Fig. 4D), suggesting that the binding site#1 was the true *miR-3591-5p* targeting site. In addition, the *miR-3591-5p* inhibition significantly enhanced the mRNA and protein expression *PRKAA2* in LPS-treated chondrocytes (Fig. 4E and H), while the *miR-3591-5p* overexpression markedly decreased the *PRKAA2* expression in LPS-treated chondrocytes (Fig. S2A and S2D). All together, the above data showed that *PRKAA2* was a direct downstream target genes of *miR-3591-5p* in chondrocytes.

To further investigate the *miR-3591-5p*/*PRKAA2* axis in OA chondrocytes biological function, we transfected *miR-3591-5p* inhibitor along or together with sh*PRKAA2* into chondrocytes treated with LPS and executed a series of rescue experiments. Our results certified that downregulation of *miR-3591-5p* promoted proliferation, suppressed apoptosis, and suppressed ECM degradation in LPS-treated chondrocyte, and these phenomena were reversed by the inhibition of *PRKAA2* (Fig. 4F, G, and H). Moreover, our data indicated that overexpression of *miR-3591-5p* inhibited proliferation, promoted apoptosis, and aggravated ECM degradation in LPS-treated chondrocytes, but the above effects were weakened by *PRKAA2* overexpression (Supplementary Fig. 2B, C, and D). Taken together, these results demonstrated that *miR-3591-5p* aggravated cartilage injury of OA by sponging *PRKAA2*.

### *FTO* inhibited LPS-induced chondrocyte damage through regulating *miR-3591-5p/PRKAA2* axis

**Fig. 5:**
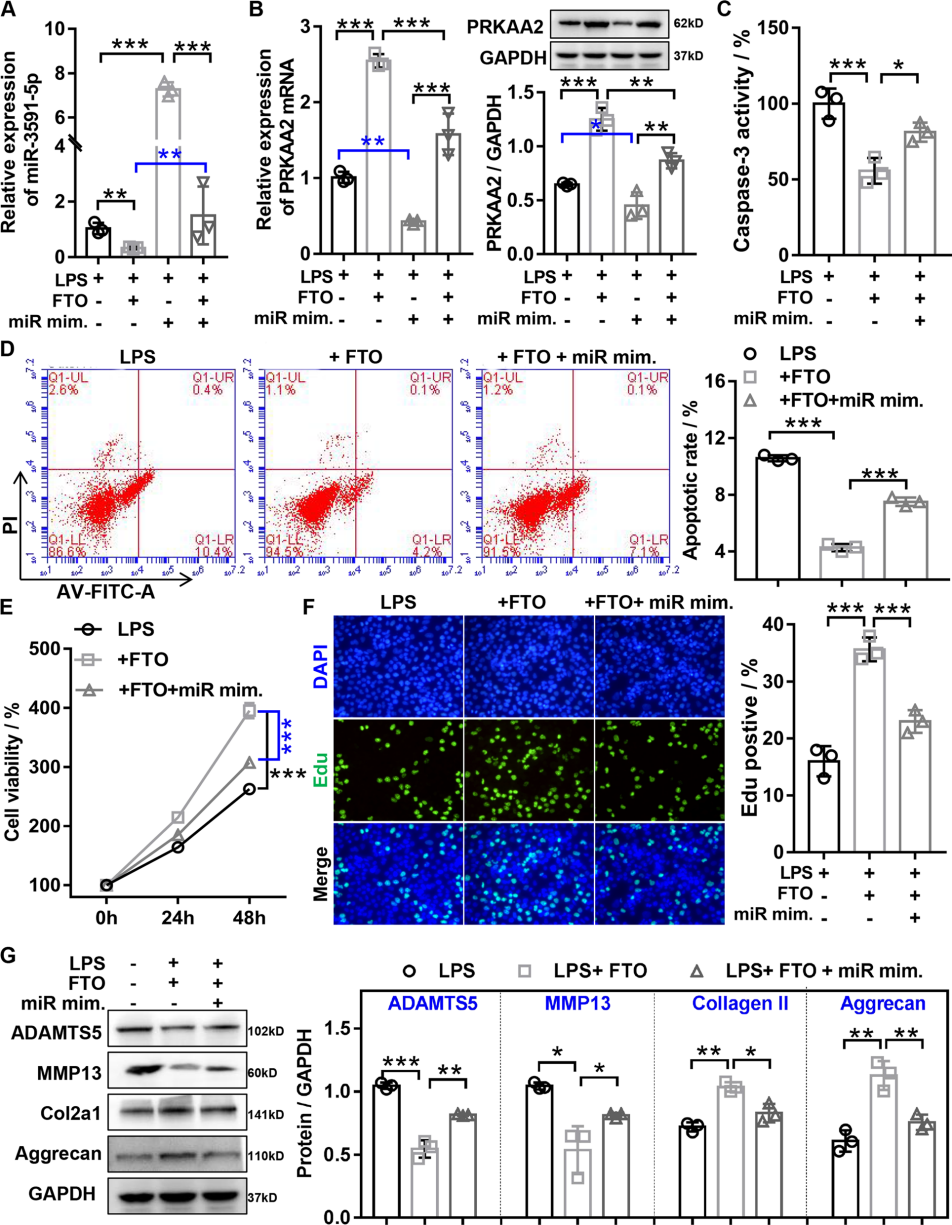
*FTO* inhibited LPS-induced chondrocyte damage through downregulating *miR-3591-5p*. Chondrocytes were transfected with *FTO* overexpression adenovirus or a combination of *FTO* overexpression adenovirus and *miR-3951* mimics and then treated with 40 ng/mL LPS for 24 h. **A** The expression of *miR-3591-5p* was assessed by RT-qPCR. **B** The mRNA and protein expression of *PRKAA2* was measured by both RT-qPCR and western blotting. **C** The caspase-3 activity was evaluated by caspase-3 activity kit. **D** The apoptosis of chondrocytes was evaluated by flow cytometry. **E** CCK-8 was detected the viability of chondrocytes. **F** EDU staining was used to assess the proliferation ability of chondrocytes. **G** Western blotting was applied to detect the expression of *MMP13*, *ADAMTS5*, *Aggrecan*, and *COL2A1*. LPS, lipopolysaccharide; miR mim., *miR-3591-5p* mimics adenovirus; *FTO*, *FTO* overexpression adenovirus; *n* = 3, *p < 0.05, **p < 0.01, ***p < 0.001

**Fig. 6:**
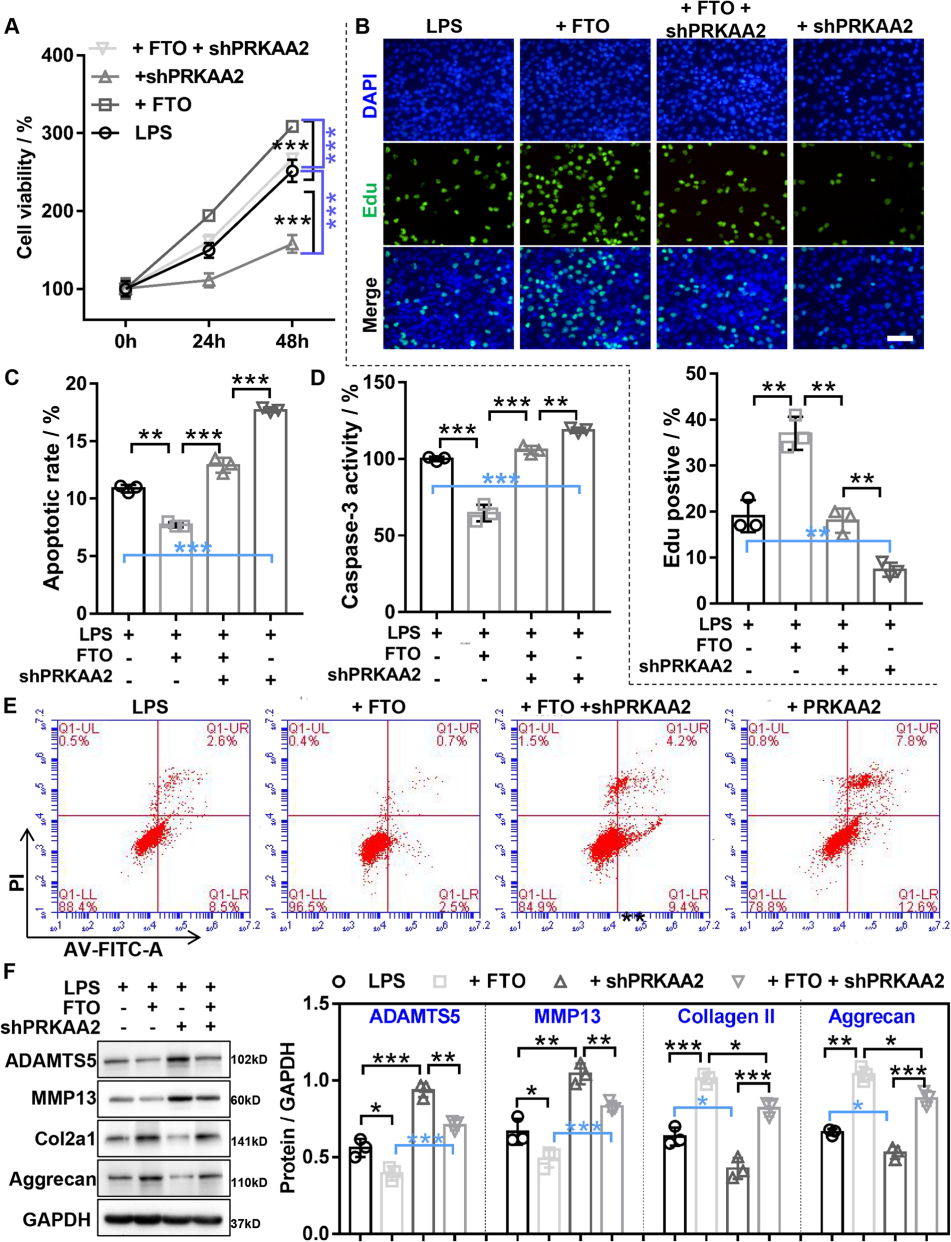
*FTO* inhibited LPS-induced chondrocyte damage through upregulating *PRKAA2*. Chondrocytes were transfected with *FTO* overexpression or sh*PRKAA2* or a combination of *FTO* overexpression and sh*PRKAA2* and then treated with 40 ng/mL LPS for 24 h. **A** CCK-8 was used to detect the viability of chondrocytes. **B** EDU staining was applied to detect the proliferation ability of chondrocytes. **D** and **F** The apoptosis of chondrocytes was assessed by flow cytometry. **E** The caspase-3 activity was measured by caspase-3 activity kit. **F** Western Blot was applied to assess the protein expression of *MMP13*, *ADAMTS5*, *Aggrecan*, and *COL2A1*. LPS, lipopolysaccharide; sh*PRKAA2*, *PRKAA2* knockdown adenovirus; *FTO*, *FTO* overexpression adenovirus; *n* = 3, *p < 0.05, **p < 0.01, ***p < 0.001

To further confirm whether *FTO* regulated *PRKAA2* expression by inhibiting *miR-3591-5p* expression, RT-qPCR and western blot disclosed that *FTO* overexpression or *miR-3591-5p* overexpression resulted in significantly increasing or decreasing *PRKAA2* expression in LPS-treated chondrocytes, but the above effect was partially weakened by co-transfection of overexpressed *FTO* and *miR-3591-5p* (Fig. 5A and B), suggesting that *FTO* increased *PRKAA2* expression by inhibiting *miR-3591-5p*. To further confirm *FTO*/*miR-3591-5p* axis regulated chondrocyte biological function by executing a series of rescue experiments, the results of caspase-3 activity assay and flow cytometry assay showed that *miR-3591-5p* overexpression could partially reverse the decreased apoptosis in LPS-treated chondrocytes mediated by *FTO* overexpression (Fig. 5C and D). The results of CCK-8 and EDU assay showed that *miR-3591-5p* overexpression could partially reverse the increased proliferation in LPS-treated chondrocytes mediated by *FTO* overexpression (Fig. 5E and F). The results of western blot indicated that *miR-3591-5p* overexpression could partially reverse the reduced ECM degradation in LPS-treated chondrocytes mediated by *FTO* overexpression (Fig. 5G). Next, we verified *FTO* inhibited LPS-induced chondrocytes damage through regulating *PRKAA2*. The results of CCK-8 and EDU disclosed that *PRKAA2* knockdown or *FTO* overexpression significantly decreased or increased the cell proliferation in chondrocytes with LPS, whereas the above effects were reversed by co-transfection of sh*PRKAA2* or *FTO* (Fig. 6A and B). Flow cytometry assay and caspase-3 activity assays showed that *PRKAA2* knockdown or *FTO* overexpression significantly enhanced or reduced the apoptosis in chondrocytes with LPS, whereas the above effects were reversed by co-transfection of sh*PRKAA2* and *FTO* (Fig. 6C, D, and E). Western blotting showed that *PRKAA2* knockdown or *FTO* overexpression significantly aggravated or restrained the ECM degradation in chondrocytes with LPS, as manifested by increased or inhibited expression of *ADAMTS5* and *MMP13*, and decreased or enhanced expression of *Collagen II* and *Aggrecan*, whereas the above effects were reversed by co-transfection of sh*PRKAA2* or *FTO* (Fig. 6F). Taken together, our data demonstrated that *FTO* inhibited LPS-induced chondrocyte damage through regulating *miR-3591-5p*/*PRKAA2* axis.

### *FTO* ameliorates the progression of OA in mice

**Fig. 7:**
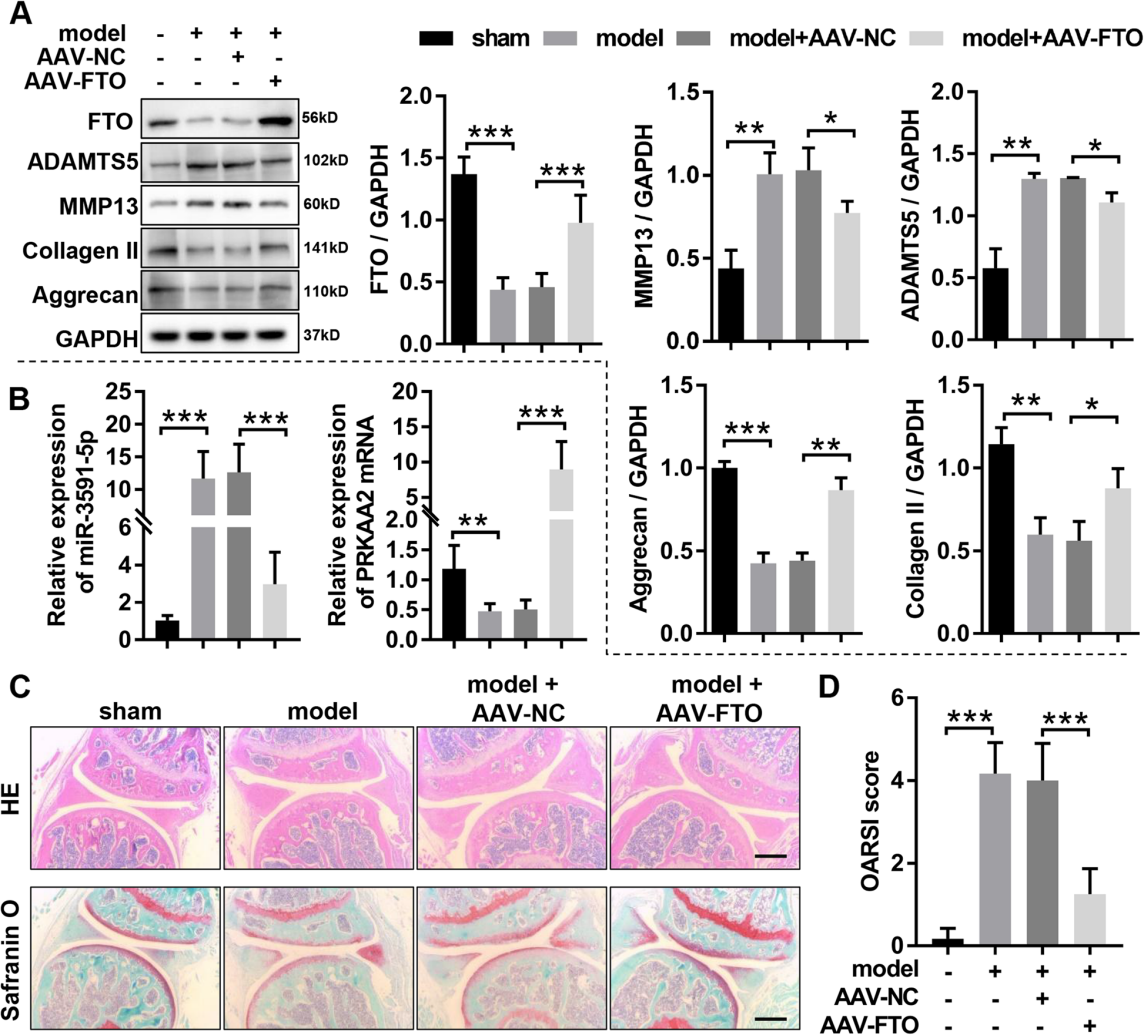
*FTO* ameliorated the OA progression in mice. **A** The protein expression of *FTO*, *MMP13*, *ADAMTS5*, *Aggrecan*, and *COL2A1* was evaluated by western blotting in the knee cartilage of OA mice with or without AAV-*FTO* treatment. **B** RT-qPCR was applied to assess the expression of *miR-3591-5p* in the knee cartilage of OA mice with or without AAV-*FTO* treatment. **C** The section of the knee joints was stained by HE staining and Safranin O & Fast Green staining, respectively. **D** OARSI scores of the knee joints in OA mice with or without AAV-*FTO* treatment. Sham, mice without DMM; model, DMM surgery-induced OA mice; AAV-NC, negative control corresponding to *FTO* overexpression adeno-associated virus; AAV-*FTO*, *FTO* overexpression adeno-associated virus; *n* = 6, *p < 0.05, **p < 0.01, ***p < 0.001

To demonstrate *FTO* affects the progression of OA in vivo, the DMM-induced OA mice were injected intra-articularly with AAV-*FTO* or AAV-NC. The results of western blotting disclosed that the *FTO* was outstandingly downregulated in articular cartilage of OA mice compared with that of in articular cartilage of mice without OA, while AAV-*FTO* treatment significantly enhanced *FTO* expression in articular cartilage of OA mice (Fig. 7A). Then, western blotting was applied to estimate the protein expression of cartilage degeneration markers (*MMP-13* and *ADAMTS-5*) and the chondrogenic markers (*Collagen II* and *Aggrecan*) in articular cartilage, and the results showed that overexpression of *FTO* obviously suppressed the expression of cartilage degeneration markers (*MMP-13* and *ADAMTS-5*) and outstandingly augmented the expression of chondrogenic markers (*Collagen II* and *Aggrecan*) in articular cartilage of OA mice (Fig. 7A). Meanwhile, the results of RT-qPCR indicated that overexpression of *FTO* significantly inhibited the *miR-3591-5p* expression in articular cartilage of OA mice, whereas markedly enhanced the expression of *PRKAA2* (Fig. 7B). Finally, we used the HE and Safranin O-fast green to investigate cartilage surface histopathological changes after intra-articular injection in OA mice with AAV-*FTO*. The results showed that OA mice showed more severe degenerative OA changes than the sham mice; these modifications were obviously ameliorated by overexpression of *FTO* (Fig. 7C). Consistent with histological analyses, OARSI scores in articular cartilage of OA mice prominently increased compared with that in articular cartilage of mice without OA, but the increased OARSI scores were obviously inhibited following overexpression of *FTO* (Fig. 7D). Taken together, our data suggested that overexpression of *FTO* alleviated OA progression and regulated *miR-3591-5p*/*PRKAA2* axis in OA mice.

## Discussion

The critical roles of m6A are increasingly important in recent years [28, 29]. The m6A modification can regulate a range of biological progression, such as maturation, alternative splicing, and mRNA translation [30]. As a ubiquitous epitranscriptome modification in most eukaryotic mRNAs, catalytic enzymes affecting m6A levels have been widely introduced, including enzymes (*METTL3*, *METTL14*, and *WTAP*) that promote increased m6A levels and enzymes (*FTO* and *ALKBH5*) that wipe off m6A from RNA [31–34]. There are few studies on their role and regulatory mechanism in OA. Liu et al. verified that *METTL3* promoted the occurrence and development of experimental OA by mediating the inflammation and apoptosis of chondrocyte [35]. A latest research showed that *METTL3* regulated autophagy-*GATA4* axis to intensify cellular senescence and OA progression by mediating m6A modification of *ATG7* [10]. However, other essential m6A regulatory enzymes have not been reported in OA yet.

Chondrocytes, as a single cell type in cartilage tissue, have a central role in maintaining cartilage damage and remodeling [36, 37]. Moreover, chondrocyte apoptosis and ECM degradation are intently related to the occurrence and development of OA [38, 39]. Hence, suppression of chondrocyte apoptosis and ECM degradation are considered to be a therapeutic target for OA. In this study, our results established that upregulation of *FTO* inhibited chondrocyte apoptosis and ECM degradation in vivo and in vitro, uncovering that *FTO* is a promising therapeutic target molecule for OA.

It has been reported that in addition to protein-coding genes, large non-coding RNAs function as gene regulators during the OA progression [40, 41]. MiRNA processing is also regulated specifically for OA. However, there are no reports exploring the biological function of m6A modification during miRNA processing in OA. In this study, our data attested that *FTO* negatively regulated the expression of *miR-3591-5p* and positively regulated *pri-miR-3591*. At the same time, we analyzed the *pri-miR-3591* sequence and found that it also contained the “RRACH” motif which has been confirmed as a highly conserved common sequence for RNA m6A modification [42, 43]. Further molecular experiments revealed that knockdown of *FTO* mediated the m6A modification of *pri-miR-3591* to promote the maturation of *miR-3591-5p* in OA. Previous studies have identified *miR-3591-5p* was markedly increased in the serum of patients with glioblastoma [44] and patients with chronic HBV infection [45]. Lu et al. demonstrated that *miR-3591-5p* is molecule required for radiation-induced *TGF-β* activation [46]. However, the biological functions of *miR-3591-5p* in OA have not been reported. Our founding demonstrated that *miR-3591-5p* aggravated LPS-induced chondrocytes damages, whereas *miR-3591-5p* knockdown obviously inhibited LPS-induced chondrocytes damages. These results implied that *miR-3591-5p* downregulated by *FTO* with m6A modification played a protective role in OA progression.

Current studies have disclosed that *PRKAA2* (also known as *AMPK*) plays a central role in regulating joint homeostasis and OA development, and targeting *PRKAA2* is expected to become a therapeutic strategy for OA [47]. *PRKAA2* knockdown in chondrocytes aggravated chondrocytes apoptosis, degradation of cartilage ECM, and inflammatory response, leading to disruption of joint homeostasis and exacerbation of OA progression [48], but *PRKAA2* activity outstandingly ameliorated the severity of knee joint cartilage destruction in mice with OA[49–51]. However, the relationship between *miR-3591-5p* and *PRKAA2* has not been explored yet. Our results uncovered that *miR-3591-5p* significantly suppressed *PRKAA2* expression by sponging *PRKAA2* in OA chondrocytes. Furthermore, our data also certified that overexpression of *PRKAA2* obviously reduced the damage of *miR-3591-5p* on OA chondrocytes, while knockdown of *PRKAA2* significantly lessened the protective effect of *miR-3591-5p* on OA chondrocytes. Interestingly, our findings also uncovered that *FTO* inhibited the LPS-induced chondrocyte damage by regulating *miR-3591-5p/PRKAA2* axis in vitro.

In conclusion, our data revealed that *FTO* alleviated OA cartilage damage by the *FTO/miR-3591-5p/PRKAA2* axis, which offered fire-new insights into the pathogenesis and therapeutic strategies for OA.

## Supplementary Information

**Additional file 1: Supplementary Figure 1.** FTO knockdown aggravated LPS-induced chondrocyte damage in vitro. **Supplementary Figure 2.** miR-3591-5p overexpression aggravated LPS-induced chondrocytes damage by inhibiting PRKAA2. **Supplementary Table 1.** The sequences of shRNAs, miR-3591-5p inhibitor/mimics, and their negative control in this study. **Supplementary Table 2.** Primers used for RT-qPCR and other assays in this study.

## Data Availability

All data are presented in this paper.
