# Supplementary material for: FTO-mediated m6A demethylation of pri-miR-3591 alleviates osteoarthritis progression
Source: Arthritis Res Ther. 2023 Apr 1;25:53. doi: 10.1186/s13075-023-03035-5 (PMC10067311; doi:10.1186/s13075-023-03035-5)
Supplement: Supplementary file 1 — Additional file 1: Supplementary Figure 1. FTO knockdown aggravated LPS-induced chondrocyte damage in vitro. Supplementary Figure 2. miR-3591-5p overexpression aggravated LPS-induced chondrocytes damage by inhibiting PRKAA2. Supplementary Table 1. The sequences of shRNAs, miR-3591-5p inhibitor/mimics, and their negative control in this study. Supplementary Table 2. Primers used for RT-qPCR and other assays in this study. [file 13075_2023_3035_MOESM1_ESM.docx]

Supplementary material

**Contents**

1. Supplementary Figures

Supplementary Fig. 1 FTO knockdown aggravated LPS-induced chondrocyte damage *in vitro*.

Supplementary Fig. 2 miR-3591-5p overexpression aggravated LPS-induced chondrocytes damage by inhibiting PRKAA2.

2. Supplementary Tables

Supplementary Table 1 The sequences of shRNAs, miR-3591-5p inhibitor/mimics, and their negative control in this study.

Supplementary Table 2 Primers used for RT-qPCR and other assays in this study.

**1. Supplementary Figures**


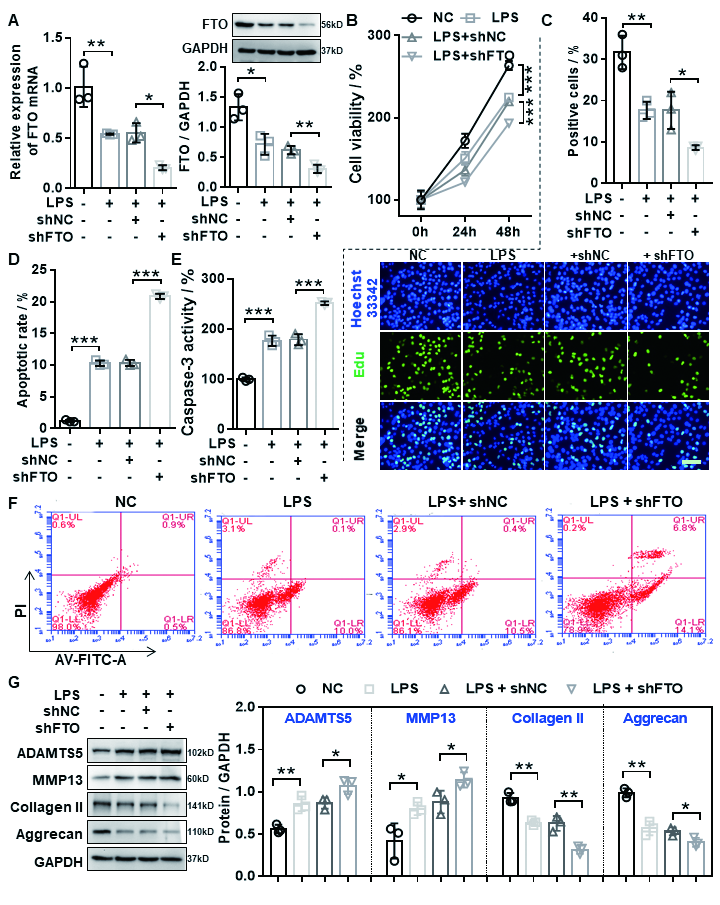


**Supplementary Fig. 1** **FTO knockdown aggravated LPS-induced chondrocyte damage *in vitro*.** Chondrocytes were transfected with shFTO or shNC, and then treated with 40 ng/mL LPS for 24 h. (**A**) RT-qPCR and western blot were carried out to confirm the infection efficiency of shFTO and shNC. (**B**) CCK-8 was assessed the viability of chondrocytes. (**C**) Edu staining was applied to evaluate the proliferation ability of chondrocytes. (**D and F**) The apoptosis of chondrocytes was assessed by flow cytometry. (**E**) The caspase-3 activity was detected using caspase-3 activity kit. (**G**) Western Blot was applied to measure the expression of MMP13, ADAMTS5, Aggrecan, and COL2A1. LPS, lipopolysaccharide; shFTO, FTO knockdown adenovirus; shNC, negative control corresponding to shFTO adenovirus; n=3, *p <0.05, ** p <0.01, *** p <0.001.


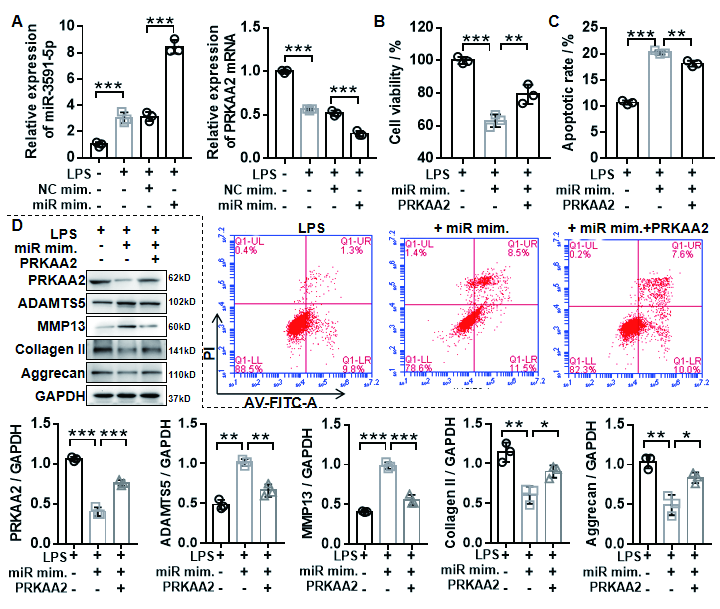


**Supplementary Fig. 2 miR-3591-5p overexpression aggravated LPS-induced chondrocytes damage by inhibiting PRKAA2**. (**A**) Chondrocytes were transfected with miR-3951-5p mimics adenovirus or NC mimics adenovirus, and then treated with 40 ng/mL LPS for 24 h. RT-qPCR was applied to assess the mRNA expression miR-3591-5p and PRKAA2. (**B**) Chondrocytes were transfected with miR-3951-5p mimics adenovirus or a combination of miR-3951-5p mimics adenovirus and PRKAA2 adenovirus, and then treated with 40 ng/mL LPS for 24 h. CCK-8 was applied to assess the viability of chondrocytes. (**C**) Chondrocytes were transfected with miR-3951 mimics or a combination of miR-3951-5p mimics adenovirus and PRKAA2 adenovirus, and then treated with 40 ng/mL LPS for 24 h. The apoptosis of chondrocytes was detected by flow cytometry. (**D**) Chondrocytes were transfected with miR-3951-5p mimics or a combination of miR-3951-5p mimics adenovirus and PRKAA2 adenovirus, and then treated with 40 ng/mL LPS for 24 h. Western Blot was used to detect the expression of MMP13, ADAMTS5, Aggrecan, and COL2A1. LPS, lipopolysaccharide; NC mim., negative control corresponding to miR-3591-5p mimics adenovirus; miR mim., miR-3591-5p mimics adenovirus; NC inh., negative control corresponding to miR-3591-5p inhibitor adenovirus; miR inh., miR-3591-5p inhibitor adenovirus; PRKAA2, PRKAA2 overexpression adenovirus; n=3, *p <0.05, **p <0.01, ***p <0.001.

**2. Supplementary Tables**

**Supplementary Table 1 The sequences of shRNAs, miR-3591-5p inhibitor/mimics, and their negative control in this study.**

| Names | Sequences (5’-3’) |
| --- | --- |
| shFTO (human) | TCACCAAGGAGACTGCTATT |
| shFTO (mouse) | CCAGGGAGACTGCTATTTCAT |
| shPRKAA2 (human) | CCCACTGAAACGAGCAACTAT |
| miR-3591 mimics (human) | sense, UUUAGUGUGAUAAUGGCGUUUGA |
|  | antisense, AAACGCCAUUAUCACACUAAAUU |
| NC mimics (human) | sense, UUGUACUACACAAAAGUACUG |
|  | antisense, GUACUUUUGUGUAGUACAAUU |
| miR-3591 inhibitor (human) | UCAAACGCCAUUAUCACACUAAA |
| NC inhibitor (human) | CAGUACUUUUGUGUAGUACAA |

**Supplementary Table 2 Primers used for RT-qPCR and other assays in this study.**

| Names | Primer sequences (5’-3’) |
| --- | --- |
| FTO | Forward, GAAGCACTGTGGAAGAAGATGGA |
|  | Reverse, GGCAAGGATGGCAGTCAAGAT |
| PRKAA2 | Forward, GTGAAGATCGGACACTACGTG |
|  | Reverse, CTGGAAGGTGGACAGCGAGG |
| pri-miR-3591 | Forward, AGAGCCAGACACAGAAGA |
|  | Reverse, TCTCTGCTTGGTCCTTGT |
| pre-miR-3591 | Forward, AGGGAAAGGCGGAAGAGA |
|  | Reverse, ATTGCCCTACCCGCCAAGAAATGTAAGGTAT |
| miR-3591-5p | Forward, ACACTCCAGCTGGG TTTAGTGTGATAATGGC |
|  | Reverse, CAGTGCGTGTCGTGGAGT |
| GAPDH | Forward, CGGCAAGTTCAACGGCACAGT |
|  | Reverse, ACGCCAGTAGACTCCACGACAT |
| U6 | Forward, CTCGCTTCGGCAGCACA |
|  | Reverse, TGGTGTCGTGGAGTCG |
| Primers used for MeRIP-qPCR | |
| Pri-miR-3591 | Forward, AACACCAGCAAGGAGAAC |
|  | Reverse, TCCTTCCCTCGTTGTCTT |
| Primiers used for in vitro transcription | |
| Pri-miR-3591 | Forward, ATTGCCTAATACGACTCACTATAGGGAGAAAC  ACCAGCAAGGAGAAC |
|  | Reverse, AAGAATTTGGGCCGGCACTGTCAGACCGA |
| Pri-miR-1-1 | Forward, ATTGCCTAATACGACTCACTATAGGGAGAGG  GCCCGGCAGAGA |
|  | Reverse, CAATAACCCCGCCTCCCCCCCCCGCC |
